# From mechanisms to management: a comprehensive review of sarcopenia in gastric cancer

**DOI:** 10.3389/fnut.2026.1726256

**Published:** 2026-01-28

**Authors:** Wenhao Liu, Hongliang Cao, Xuanpeng Zhou, Luanbiao Sun, Linchun Li, Xinyuan Song, Yang Gao, Jianpeng Xing, Shuohui Gao

**Affiliations:** 1 China-Japan Union Hospital of Jilin University, Changchun, Jilin, China; 2 Department of Urology II, The First Hospital of Jilin University, Changchun, Jilin, China; 3 Department of Statistics, The Chinese University of Hong Kong, New Territories, Hong Kong, China; 4 Zhalute Banner People’s Hospital, Tongliao, Inner Mongolia, China

**Keywords:** gastric cancer, management, mechanism, prognosis, sarcopenia

## Abstract

Gastric cancer (GC), a leading cause of global cancer mortality, induces systemic changes impacting patient prognosis. A growing body of evidence shows a significantly increased prevalence of sarcopenia in GC patients, closely linked to poor outcomes such as higher postoperative complications, enhanced chemotherapy toxicity, and shortened survival. However, its underlying mechanisms and optimal management remain not fully clarified. This review comprehensively analyzes the pathological mechanisms and clinical significance of GC-related sarcopenia, emphasizing systemic inflammation, metabolic/nutritional disorders, neuroendocrine dysfunction, and anti-tumor therapy impacts. Additionally, feasible management methods such as nutritional support, exercise intervention, and related drug treatment were also proposed. By synthesizing current evidence, we delineate sarcopenia’s integral role in GC and propose pragmatic strategies to ultimately improve patient outcomes.

## 1 Introduction

Gastric cancer (GC) is a malignant tumor that originates from the cells of the gastric mucosa, with gastric adenocarcinoma being the most common. Globally, although the overall age-standardized incidence rate has decreased, the total number of GC cases has increased annually (1). However, due to its high heterogeneity and concealment, diagnosis is often at an advanced stage, and treatment requires multidisciplinary collaboration (2). With the continuous progression of GC, the consumption of skeletal muscle and body fat leads to a significant decrease in weight, resulting in the appearance of cachexia (3). This imposes a huge economic burden on society and impairs patients’ life span and quality of life.

Sarcopenia was initially defined as a disorder characterized by low muscle mass accompanied by reduced muscle strength or impaired physical function. While it is most commonly linked to advancing age, secondary sarcopenia induced by multiple etiologies also exists, and the condition markedly elevates the risks of disability and mortality (4). Clinically, sarcopenia is featured by insidious onset and progressive progression, with its early manifestations often misattributed to natural aging, including typical signs such as gradual reduction in muscle-associated limb (particularly lower limb) circumference and unexplained weight loss, while manifestations related to declining muscle strength primarily center on impaired physical mobility, encompassing reduced static grip strength, slowed and shortened gait, and compromised balance ability (4, 5). Notably, sarcopenia is further associated with a spectrum of adverse health outcomes: it not only significantly increases the risks of falls and fractures, which in turn lead to loss of activities of daily living (ADL) ability, but also serves as an independent predictor of elevated all-cause mortality (5).

Emerging evidence suggests that the incidence of sarcopenia in GC patients is significantly higher than that in the general population, attributed to abnormal tumor metabolism, surgical trauma, inadequate nutritional intake, and persistent inflammatory states (6). On the other hand, sarcopenia can seriously affect the treatment prognosis of patients with GC and is a core issue that urgently needs attention in the comprehensive clinical management of GC (7).

Sarcopenia, related to GC, has attracted widespread attention and has become a key issue to be solved. This article systematically reviews the epidemiological association between the two, integrates multidimensional mechanisms, and summarizes the potential diagnosis and treatment system, with the aim of providing new ideas and methods for improving the therapeutic effect and quality of life of patients with GC by focusing on and intervening in sarcopenia.

## 2 Epidemiological characteristics and clinical impacts of sarcopenia in GC

Existing research systems have revealed that sarcopenia is a widespread and significantly heterogeneous clinical phenomenon among patients with GC. Its incidence varies depending on diagnostic criteria, tumor stage, and population characteristics, but consistently correlates with poor prognosis. More importantly, sarcopenia is not only a potential risk predictor of GC, but also significantly increases the risk of surgical complications and weakens the tolerance and efficacy of chemotherapy. Its role runs through the entire process of GC diagnosis and treatment. A summary of key studies investigating the prevalence and outcomes of sarcopenia in GC patients is presented in Table 1.

**Table 1:** Prevalence and clinical outcomes of sarcopenia in GC.

Author (year)	Country/region	Research subjects	Sample size	Diagnostic criteria	Main outcomes
Wagh et al. (12) (2024)	India	Operable GC	74	CT (L3 SMI)	Sarcopenia prevalence: 43.2%; no significant association with early postoperative complications
Xiong et al. (13) (2022)	China	GC (all stages)	225	CT (muscle mass) + serum IL-16	Sarcopenia prevalence: 47.5%; sarcopenia + high IL-16 predicts poorest OS/RFS
Wu et al. (8) (2021)	China	Post-radical gastrectomy	210	AWGS2019 vs. EWGSOP2	Higher detection by AWGS2019 (20.5% vs. 11.3%); both predict OS, AWGS superior
Kim et al. (9) (2023)	South Korea	Gastrectomy patients	362	DXA (whole body) + CT (L3)	CT/DXA-diagnosed sarcopenia increases complications; CT shows higher risk
Sun et al. (16) (2022)	China (Taiwan)	Asian population (incl. GC)	77,608 (GC = 317)	Muscle mass/strength assessment	Sarcopenia increases GC risk (HR = 2.25, 95% CI: 1.54–3.23)
Kanbur et al. (10) (2025)	Turkey	Metastatic GC	177	CT (SMI) + SII/PNI inflammatory indices	Sarcopenia prevalence: 71.8%; SII/PNI + sarcopenia predict reduced survival
Ding et al. (11) (2022)	China	Locally advanced GC	134	SII + PNI combined screening	Sarcopenia prevalence: 23.13%; SII + PNI improves screening/prognosis accuracy
Li et al. (22) (2025)	China	Locally advanced GC (gastrectomy)	198	CT (L3 SMI); SML (sex-specific SMI changes)	Preoperative (23.7%)/postoperative sarcopenia (33.3%) and perioperative SML (HR = 11.231) all independently predict worse OS
Kudou et al. (14) (2017)	Japan	EGJC/UGC	358	CT (L3 SMI)	Sarcopenia rates: EGJC 32.2%, UGC 25.8%; reduces 5-year OS/RFS
Sierzega et al. (18) (2019)	Poland	Western GC (gastrectomy)	214	CT (L3 SMI)	Total complications (43% vs. 23%), severe complications, and reoperation rates
Uchida et al. (20) (2021)	Japan	GC (gastrectomy)	353	CT (IMAC + muscle mass)	Low muscle mass + high IMAC increases infection risk (OR = 3.2, *p* < 0.01)
Olmez et al. (21) (2021)	Turkey	GC Surgery	152	CT (SMI)	Surgical site infection (29.8% vs. 11.9%, *p* = 0.003)
Chen et al. (25) (2019)	China	Laparoscopic gastrectomy	286	CT (L3 SMI)	Independent risk for complications (OR = 2.8, *p* = 0.004)
Umemoto et al. (23) (2025)	Japan	GC (gastrectomy)	255	Bioelectrical impedance	Preoperative sarcopenia:19.2%; 18.4% new-onset postoperative sarcopenia; both link to shorter OS
Bir et al. (24) (2023)	Turkey	Stage II/III GC (gastrectomy)	84	CT (L3 SMI, AWGS2019)	Sarcopenia:36.9%, OS 14 vs48m (*p* < 0.001); independent OS predictor (HR = 3.31)

GC, gastric cancer; CT, computed tomography; SMI, skeletal muscle index; SML, skeletal muscle loss; IL-16, interleukin-16; AWGS2019, Asian Working Group for Sarcopenia 2019; EWGSOP2, European Working Group on Sarcopenia in Older People 2; DXA, dual-energy x-ray absorptiometry; HR, hazard ratio; CI, confidence interval; SII, systemic immune-inflammation index; PNI, prognostic nutritional index; OS, overall survival; RFS, recurrence-free survival; EGJC, esophagogastric junction carcinoma; UGC, upper gastric cancer; IMAC, intramuscular adipose tissue content.

### 2.1 Heterogeneity in the incidence of sarcopenia in GC

The incidence of sarcopenia in the GC population shows significant fluctuations. This difference is not random but is jointly regulated by three core factors. From the perspective of diagnostic criteria, the differences among various guidelines directly lead to deviations in the calculation of incidence rates. A comparison of the diagnostic criteria from the Asian Working Group for Sarcopenia 2019 (AWGS2019) and the European Working Group on Sarcopenia in Older People 2 (EWGSOP2) revealed that the former yielded a higher detection rate (20.5% vs. 11.3%) in patients who underwent radical gastrectomy for GC and exhibited superior predictive performance for overall survival (OS) (8). Further research suggests that the AWGS standard is more applicable to male GC patients in Asia (9). This discovery has broken the perception that a single diagnostic criterion is applicable to all populations, emphasizing that the construction of regional and population-specific diagnostic models is the key to improving the accuracy of sarcopenia detection and also the prerequisite for subsequent personalized clinical intervention.

From the perspective of tumor stage and location, the incidence rate shows a significant increasing trend as GC progresses. A study in Turkey involving 177 patients with metastatic GC showed that the incidence of sarcopenia was as high as 71.8%, significantly higher than 23.13% for locally advanced GC (10, 11). Combined systemic immune-inflammation index (SII) and prognostic nutritional index (PNI) scores as a screening marker for sarcopenia in patients. Even in early operable GC, the detection rate in the Indian study reached 43.2% (12). The research findings of Xiong et al. (13) have provided another piece of evidence for the prognostic value of sarcopenia in patients with GC. They observed that the incidence of sarcopenia in patients with GC reached 47.5%, and when sarcopenia coexists with high expression of the pro-inflammatory factor IL-16, the prognosis of patients is the worst. At the tumor site level, the incidence of sarcopenia in patients with esophagogastric junction cancer (EGJC) (32.2%) is higher than that in patients with upper gastric cancer (UGC) (25.8%), and the 5-year OS and recurrence-free survival (RFS) of both types of patients are shortened due to sarcopenia (14).

From a population perspective, a global meta-analysis of solid tumor patients provides crucial evidence for regional differences (15). The overall prevalence of sarcopenia among patients with solid tumors worldwide is 35.5%, but it is significantly higher in Europe and America than in Asia. Compared with Europe and America, the Asian population shows a stronger “risk association” in the field of GC. A cohort study of an Asian population showed that the risk of GC in patients with sarcopenia was 2.25 times that of those without sarcopenia (95% CI: 1.54–3.23) (16). Meta-analysis of Asian GC patients further confirmed that the overall incidence of sarcopenia was 26.6%, and old age, high nutritional risk, and large tumor volume were independent risk factors (17). This suggests that in the Asian region where GC is highly prevalent, sarcopenia may serve as an early risk screening indicator, providing a new target for the primary prevention of GC.

### 2.2 The negative impact of sarcopenia on the surgical treatment outcome of GC

Surgical treatment is the first choice for patients with resectable GC, while conversion surgery can also be performed for some unresectable patients according to their individual conditions to reduce tumor load and improve the quality of life of patients. Many studies have shown that sarcopenia is closely related to postoperative complications and long-term survival in patients with GC.

The risk effect of sarcopenia on short-term complications after GC surgery is both universal and specific. On the one hand, a Western study on GC patients showed that the total postoperative complication rate of those with sarcopenia before surgery was 43%, nearly twice that of those without sarcopenia (23%), and the rates of severe complications (Clavien–Dindo ≥3a) and reoperation were also significantly increased (18). Meta-analysis of the Asian population further quantified this risk. The results showed that sarcopenia could increase the risk of postoperative complications in GC patients by nearly three times, with particularly significant elevated effects on the risks of pneumonia (RR = 2.64) and obstruction (RR = 3.96) (19). The association between sarcopenia and infectious complications is particularly prominent. Uchida et al. (20) found that GC patients with sarcopenia combined with elevated intramuscular fat content had a three times higher risk of postoperative infection than those without this condition. Research in Turkey also confirmed that the surgical site infection rate of GC patients with sarcopenia was 29.8%, significantly higher than that of patients without sarcopenia (11.9%, *p* = 0.003) (21).

The negative impact of sarcopenia on postoperative survival of GC shows a high degree of consistency in studies at different disease stages and with different population characteristics. Among patients with locally advanced gastric cancer (LAGC), the OS and disease-free survival (DFS) of postoperative sarcopenia were worse than those without sarcopenia (22). What is more notable is that the risk effect of perioperative muscle loss (HR = 11.231, *p* = 0.002) far exceeded the adverse effects at other stages. In another postoperative population for GC, the OS of patients with newly developed sarcopenia before and after surgery was shorter than that of the disease-free group, and the negative impact on survival was comparable between the two groups (23). Among patients with stage II/III GC, those with postoperative sarcopenia had a significantly shortened OS (48 months vs. 14 months, *p* < 0.001), which was also an independent influencing factor for OS (24).

It is worth noting that previous conclusions on sarcopenia and postoperative risks were mostly based on patients undergoing open surgery, which had limitations in the research subjects. However, a prospective study overturned the assumption that “minimally invasive surgery can avoid the risk of sarcopenia,” confirming that even with laparoscopic surgery, sarcopenia still significantly increases the risk of postoperative complications (OR = 2.752), prolongs hospital stays and increases medical costs (25).

### 2.3 The constraints of sarcopenia on the tolerance and efficacy of chemotherapy for GC

For patients with advanced unresectable GC or those requiring adjuvant chemotherapy after surgery, sarcopenia not only increases the risk of chemotherapy toxicity but also indirectly weakens the therapeutic effect by affecting the dose intensity, becoming a key factor restricting the benefits of chemotherapy. Its dynamic changes can also serve as a monitoring indicator for therapeutic efficacy and prognosis.

Firstly, sarcopenia significantly increases the risk of chemotherapy toxicity, and this risk persists throughout the entire process. At the baseline level, the incidence of sarcopenia in patients with advanced GC who received XELOX/SOX (S-1/capecitabine plus oxaliplatin) chemotherapy increased from 46.2% before treatment to 51.3%, and the risk of neutropenia increased in those with decreased skeletal muscle index (SMI) regardless of whether sarcopenia was present at baseline (26). From a mechanistic perspective, muscle mass loss may lead to a decrease in drug clearance rate and an increase in blood drug concentration, thereby exacerbating toxic reactions. This also suggests a preliminary association between low muscle mass and chemotherapy toxicity. Among patients with metastatic GC, the incidence of severe hematotoxicity (63.3% vs. 32.0%) and non-hematotoxicity (66.7% vs. 36.7%) in those with low muscle mass receiving first-line palliative chemotherapy was nearly twice that of those with high muscle mass (27).

Secondly, sarcopenia indirectly reduces the therapeutic efficacy and survival benefits by influencing the intensity of chemotherapy doses. A Japanese study has shown that among unresectable GC patients receiving 5-fluorouracil chemotherapy, those with low SMI have significantly shortened OS and progression-free survival (PFS), and low SMI is an independent adverse prognostic factor (28). This influence is even more prominent in postoperative adjuvant chemotherapy. Patients with stage II/III GC who had a low psoas major muscle index (PMI) before surgery had a lower relative dose intensity (RDI) when receiving S-1 adjuvant chemotherapy, and insufficient RDI was directly related to shortened RFS (29). Another study further confirmed that preoperative low SMI is an independent predictor that patients with GC cannot complete postoperative S-1 monotherapy adjuvant chemotherapy (30).

It is worth noting that the dynamic changes in muscle mass during neoadjuvant chemotherapy (NAC) have greater prognostic value. Suggesting that muscle mass should be dynamically monitored throughout the chemotherapy process, and the drug dose should be adjusted according to the changes in muscle mass to balance the therapeutic effect and toxicity. A retrospective study by Juez et al. (31) found that the incidence of sarcopenia in patients with locally advanced GC who received NAC was 15% higher than that in those who did not receive such (*p* = 0.048). In addition, patients with muscle mass loss of more than 6.9% during NAC had significantly worse postoperative OS and RFS than those with less loss (32, 33).

Despite this, most of the above-mentioned studies were observational ones, and there were still many confounding factors. The causality between sarcopenia and chemotherapy in gastric cancer patients still needs to be further verified by prospective studies. The impact of sarcopenia on toxicity and survival outcomes in GC patients undergoing various chemotherapy regimens is detailed in Table 2.

**Table 2:** Impact of sarcopenia on chemotherapy outcomes in GC.

Author (year)	Country/region	Research subjects	Sample size	Regimens	Main outcomes
Chan et al. (27) (2024)	Hong Kong, China	Advanced GC	158	Platinum/fluoropyrimidine-based	Low SMI independently predicted severe toxicity (OR 2.71, *p* = 0.007) and shorter OS (median OS 8.7 vs. 12.6 months, *p* = 0.001). Higher hematological and non-hematological toxicity
Li et al. (26) (2024)	China	GC (adjuvant)	39	XELOX/SOX	Significant SMI reduced (36.00 → 34.99 cm^2^/m^2^, *p* < 0.001); sarcopenia rate 46.2 to 51.3%; >5% SMI loss linked to higher grade ≥3 neutropenia (70.0% vs. 36.7%, *p* = 0.007)
Matsunaga et al. (28) (2021)	Japan	Unresectable GC	83	5-FU-based first line	Low SMI associated with higher grade 3/4 toxicity (*p* = 0.028) and shorter OS (13.8 vs. 17.3 months, *p* = 0.008). Independent prognostic factor for OS (HR 1.73, 95% CI 1.11–2.69, *p* = 0.015)
Juez et al. (31) (2024)	Spain	Locally advanced GC	61	Neoadjuvant (76% FLOT)	Significant SMM reduction during NAC (mean −2.5%, *p* < 0.001), greater in males (−10.55%). NAC group had 15% higher pre-surgical sarcopenia vs. surgery-only group (*p* = 0.048)
Sato et al. (32) (2024)	Japan	GC (neoadjuvant)	50	S-1 + cisplatin	64% had muscle loss (ΔSMI −3.4%); muscle loss ≥6.9%: independent predictor of worse OS (HR = 11.53) and RFS (HR = 4.54) post-R0 resection
Miyatani et al. (33) (2019)	Japan	Stage II/III GC	95	Adjuvant S-1	Not sarcopenia*-*focused. Delay S-1 initiation (>32 days: HR 2.44, *p* = 0.007) and RDI <70% (HR 2.37, *p* = 0.009) independently predicted worse RFS
Fujita et al. (29) (2023)	Japan	Stage II/III GC	124	Adjuvant S-1	RDI <80% (HR 3.07, *p* = 0.007) and 1-year PMI reduction rate >10% (HR 2.69, *p* = 0.024) independently predicted worse OS
Nakabayashi et al. (30) (2025)	Japan	Stage II/III GC	53	Adjuvant S-1	Preoperative low PMI independently predicted S-1 non-completion (HR 3.563, *p* = 0.030). Completion group had longer OS (*p* = 0.043)

GC, gastric cancer; SMI, skeletal muscle index; OS, overall survival; CI, confidence interval; SMM, skeletal muscle mass; NAC, neoadjuvant chemotherapy; RFS, recurrence-free survival; HR, hazard ratio; XELOX, capecitabine and oxaliplatin; SOX, S-1 and oxaliplatin; RDI, relative dose intensity; PMI, psoas muscle index; IMAC, intramuscular adipose tissue content; OR, odds ratio; FLOT, fluorouracil, leucovorin, oxaliplatin, and docetaxel.

## 3 Potential mechanisms of interaction between multiple aspects of sarcopenia and GC

The interaction between GC and sarcopenia is multi-dimensional, involving mechanisms such as nutrient depletion, inflammatory drive, therapeutic damage, and immune disorders. These mechanisms together lead to decreased treatment tolerance, increased complications and shortened survival of patients, suggesting the importance of preoperative muscle status assessment and nutritional intervention.

### 3.1 Inflammation-mediated myostasis and tumor progression

Inflammation is the core link between GC and sarcopenia. The core logic lies in the formation of a bidirectional cycle where “inflammation intensifies muscle breakdown and muscle reduction amplifies inflammation.”

A class of heterogeneous signaling molecules generated and secreted by skeletal muscle cells are collectively referred to as myokines, which participate in metabolism and inflammatory regulation through autocrine, paracrine and endocrine functions (34). In the cachexia state of GC, the balance of muscle factors is completely disrupted. IL (interleukin)-6 exhibits a “bidirectional” effect. IL-6 released by local muscles can modulate muscle metabolism via anti-inflammatory effects. However, elevated circulating IL-6 levels in GC patients accelerate myoprotein degradation by activating the JAK/STAT pathway (35). Meanwhile, by activating the STAT3 signal and inhibiting the IGF-1/Akt/mTOR pathway to reduce muscle protein synthesis, it becomes a “direct driver” of muscle atrophy (36). In addition, IL-8 actively secreted by tumor cells and immune cells not only acts as a chemokine to recruit lymphocytes and neutrophils, exacerbating local inflammation, but may also indirectly accelerate tumor progression by promoting angiogenesis (37). Persistent inflammation further amplifies muscle breakdown by activating the ubiquitin-proteasome system (UPS) (38).

The autophagy-lysosome pathway (ALP) and the UPS are the two most important ways for the body to repair or eliminate abnormal proteins. Proinflammatory factors, such as TNF-α, secreted by tumor cells and infiltrating immune cells activate the NF-κB signaling pathway, leading to excessive activation of the above two pathways. Regulating atrophy genes (including key UPS molecules), directly driving muscle protein degradation by boosting calpain and E3 ubiquitin ligase expression, and activating FoxO transcription factors to enhance ALP function are all roles of NF-κB (36, 39). Zhang’s et al. (40) research further confirmed that the expression of ALP and UPS markers in the muscles of patients with cachexia in GC was significantly increased, and it was positively correlated with the reduction of muscle fiber area (*p* < 0.05), providing clinical evidence for this mechanism.

**Figure 1:**
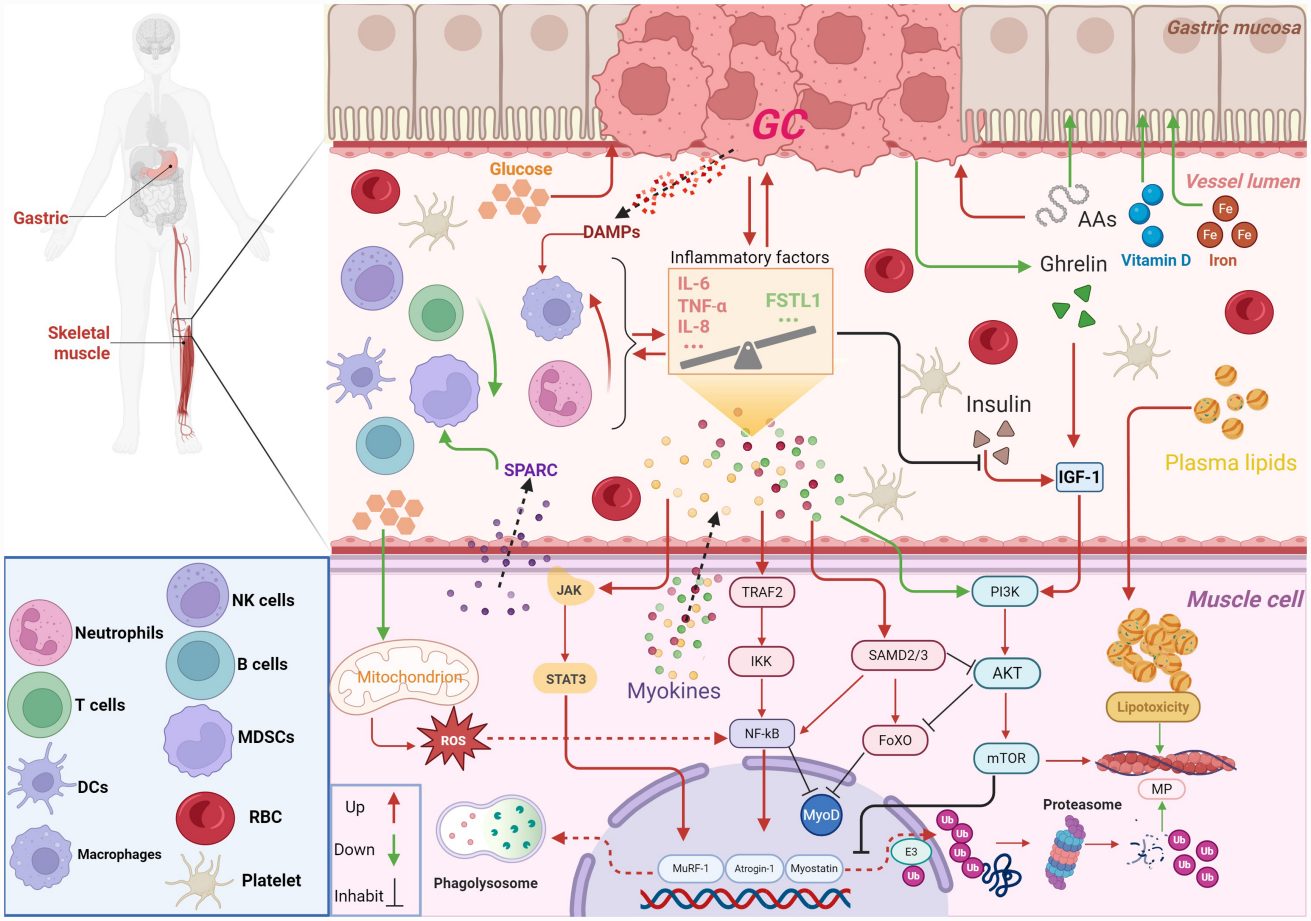
Bidirectional molecular crosstalk between GC and sarcopenia. GC promotes muscle waste by releasing inflammatory cytokines, such as IL-6, TNF-α, and IL-8, along with DAMPs, and by competing for nutrients, including glucose and amino acids. These processes collectively activate proteolytic pathways, for example MuRF-1 and Atrogin-1 via JAK/STAT3 and NF-κB signaling, and inhibit anabolic signaling, such as the IGF-1/PI3K/AKT/mTOR axis, leading to muscle atrophy. Conversely, skeletal muscle secretes myokines, exemplified by SPARC, and metabolites that feedback to modulate tumor growth and the immune microenvironment. AKT, protein kinase B; FoXO, forkhead box transcription factor; IL, interleukin; FSTL, follistatin-like protein 1; MP, muscle protein; mTOR, mammalian target of rapamycin; MuRF-1, muscle RING-finger protein-1; NF-kB, nuclear factor-kappa beta; STAT3, signal transducer and activator of transcription 3; TNF, tumor necrosis factor; TGF-α, transforming growth factor-α; GC, gastric cancer; AAs, amino acids; SPARC, secreted protein acidic and rich in cysteine; IGF-1, insulin-like growth factor 1; PI3K, phosphoinositide 3-kinase; Ub, ubiquitination; DAMPs, damage-associated molecular patterns; JAK, janus kinase; TRAF2, TNF receptor associated factor 2; IKK, IκB kinase; SAMD, SMAD family member; MyoD, myoblast determination protein; Atrogin-1, muscle atrophy f-box protein; RBC, red blood cell; MDSCs, myeloid-derived suppressor cells; ROS, reactive oxygen species. Created in BioRender. Liu, W. (2026) https://BioRender.com/o139s2c.

It is worth noting that there are not only “destructive” factors in the muscle factor network. Follicle-statin-like protein 1 (FSTL1), as an endogenous protective muscle factor, has the core function of maintaining muscle homeostasis and vascularization. Once its expression decreases, the self-repair and functional maintenance capabilities of the muscles will be directly impaired. Clinical research has further verified the pathological significance of FSTL1. The content of FSTL1 in the skeletal muscle of patients with cachexia of GC is significantly lower than that of cancer patients with stable weight (41). The complex molecular crosstalk between GC and sarcopenia is summarized in Figure 1.

### 3.2 Impaired immune function and decreased anti-tumor ability

Muscle tissue is the main metabolic organ of the body and can provide amino acids and other nutrients through catabolism. Its reduction directly results in insufficient nutrient substrates (such as glutamine) for immune cells. Research has found that the creatinine/cystatin C ratio (CCR), which reflects muscle mass and amino acid metabolism, is positively correlated with skeletal muscle index (SMI). When GC patients with low CCR receive PD-1 inhibitor treatment, the objective response rate (ORR) and disease control rate (DCR) are significantly reduced (42), indicating that muscle mass regulates the metabolic demands of immune cells. It directly affects the response to immunotherapy. Further examination shows that the neutrophil/lymphocyte ratio (NLR), platelet/lymphocyte ratio (PLR), and other inflammatory indicators in patients with GC accompanied by sarcopenia are significantly elevated, while nutritional immune indicators like PNI are decreased (10, 43).

This implies inadequate immunoglobulin synthesis and a reduced number of cytotoxic T cells (CD8^+^ T cells). As the core effector cells of anti-tumor immunity, the reduction of CD8^+^ T cells directly weaken the tumor killing ability, while the abnormality of PD-1/PD-L1 pathway aggravates the immune escape. Sarcopenia may impair the body’s immune surveillance and response to GC cells by reducing the infiltration of these immune cells in the tumor microenvironment. The research findings have proved this theory. In GC patients with sarcopenia, the number of CD8^+^ cytotoxic T cells and PD-1^+^/PD-L1^+^ immune checkpoint positive cells in tumor tissues were significantly reduced (all *p* < 0.05) (44).

The loss of muscle also disrupts the initiation conditions of anti-tumor immunity at the local structural level. Tertiary lymphoid structures (TLS) are the “functional hubs” of local immune responses in tumors, providing sites for the aggregation and activation of immune cells. Their density directly determines the efficiency of local immunity. Research has found that in GC patients with a low rate of change in psoas major muscle index (PMI), the density of TLS in tumor tissues is reduced, and the number of tumor-infiltrating lymphocytes (TILs) is decreased (45).

From the perspective of cellular function, insufficient metabolism and structural disintegration will further cause functional imbalance in immune cell subsets. CD3^+^/CD4^+^ helper T cells are the “regulatory centers” of immune homeostasis. They maintain immune homeostasis by assisting B cell activation and promoting the proliferation of cytotoxic T cells. However, cachexia in GC can induce “biased immunosuppression,” causing a pathological shift in the direction of the body’s immune response (46). Inhibiting the Th1-type response against tumors leads to an imbalance in the ratio of helper T cells to CD8^+^ cytotoxic T cells, ultimately creating space for tumor immune escape. Studies have shown that in patients with GC, elevated levels of CD3^+^/CD4^+^ cells are associated with better overall survival (OS) (46). However, when combined with sarcopenia, the levels of CD3^+^/CD4^+^ cells in patients significantly decreased, and the risk of postoperative progression was correspondingly increased.

In addition, sarcopenia can further amplify immune deficiency through the disorder of inflammatory factors. The levels of TNF-α and its receptor TNFR2 in the circulation of cachexia mice were significantly elevated (47). This signaling axis not only drives chronic inflammation but also targets regulatory T cells (Tregs) to enhance immunosuppression. The proportion of high expression of IL-16 in patients with sarcopenia (55.7% vs. 37.3%) was higher, and it was associated with poorer OS and recurrence-free survival. This was achieved by recruiting immunosuppressive cells such as macrophages and directly inhibiting anti-tumor immunity (13). Muscle mass reduction also passively loses the molecular support for immune protection. SPARC can enhance immune surveillance by inhibiting myeloid suppressor cells (MDSC) and promoting dendritic cell maturation (48) and muscle-derived SPARC also has the ability to inhibit tumor progression (49). Clinical studies have confirmed that patients with digestive tract cancer who have low SPARC levels have an increased risk of death (HR = 2.25) (50). However, SPARC derived from different tissues and cells have heterogeneous effects on the immune microenvironment. The causal relationship between myogenic SPARC and sarcopenia needs to be further verified in GC population.

More importantly, sarcopenia and tumors do not have a unidirectional influence but form a vicious closed loop of “mutual promotion of deterioration.” Tumor cells can promote the secretion of atrophic factors such as SERPINF1 and TNFRSF11B by highly expressing the transcription factor C/EBPβ (51). These factors directly activate muscle degradation pathways to exacerbate muscle atrophy, simultaneously inhibit the development and activation of immune cells, thereby undermining immune defense.

### 3.3 Malnutrition and disorders of energy metabolism

GC destroys the normal physiological structure of human nutrition absorption, resulting in the shortage of raw materials for muscle metabolism, laying a pathological foundation for subsequent energy metabolism disorders and muscle mass loss, and becoming a prerequisite for the occurrence of sarcopenia.

From the perspective of intake, GC directly leads to the decrease of active food intake by patients through the triple pathways of compression of tumor occupying, cancer pain or obstruction of food pathway (52). When exogenous nutrition cannot meet the body’s needs, the body is forced to consume skeletal muscle as an “emergency reservoir” of energy and amino acids. At this time, muscle mass loss has become a passive selection to maintain basal metabolism. In addition, inflammatory factors (such as TNF-α and IL-6) released by tumors can act on the hypothalamic appetite center through the blood–brain barrier and inhibit the active feeding drive of patients (53).

From the perspective of absorption, gastrectomy (especially radical total gastrectomy) further aggravates nutritional disorders. Surgery reduces the secretion of gastric acid and pepsin and other digestive juices, while significantly reducing the area of nutrient absorption due to the disappearance of shortening of gastric cavity, leading to malabsorption of protein, energy and trace elements such as iron and vitamin B12 (54). Iron is involved in myoglobin synthesis and vitamin B12 regulates methionine metabolism (55). Deficiency of iron and vitamin B12 may directly affect the structural stability of muscle fiber and the efficiency of muscle protein synthesis, suggesting that specific nutrients may be the key mediators linking GC and sarcopenia. Comparison of clinical data showed that the incidence of postoperative anemia, vitamin B12 deficiency, and the risk of sarcopenia in patients with total gastrectomy were higher than those in patients with proximal gastrectomy (*p* < 0.001) (56).

Tumor cell proliferation requires a large amount of energy, which results in an elevation of the body’s overall metabolic rate. Insufficient nutrient intake forms a negative energy balance, which together aggravates the contradiction between supply and demand. Crucially, tumors preferentially plunder glucose and amino acids through the “Warburg effect,” while releasing metabolites such as lactate to inhibit the mitochondrial function of muscle cells (57). This means that the body under dual pressure must activate the UPS to accelerate muscle decomposition by inhibiting mTOR and other muscle synthesis pathways to maintain energy homeostasis (40).

In addition, metabolic disorders of muscle cells themselves further amplify the damage. Carnitine, as a key metabolic factor for skeletal muscle storage, its core function is to transport long-chain fatty acids to mitochondria for oxidation through CPT1/2 enzymes and regulate the ratio of acyl-coenzyme A to maintain the activity of sugar and amino acid metabolism enzymes (58, 59). Serum carnitine as a biomarker of sarcopenia and nutritional status in preoperative gastrointestinal cancer patients. Carnitine deficiency means that muscle cells cannot effectively use fat for energy and can only rely on muscle protein breakdown for energy. However, the serum carnitine level of GC patients with sarcopenia is significantly reduced, which is not only due to the atrophy of storage pool caused by muscle mass loss, but also related to the efficiency of tumor inhibition of fatty acid oxidation (58).

Notably, tumors also affect muscle mass through the regulation of fat metabolism. In visceral fat of patients with cachexia of GC, genes related to energy metabolism, fat browning (such as uncoupling protein 1) and lipogenesis are significantly downregulated (60), suggesting that the energy supply capacity of adipose tissue is decreased. At the same time, intelectin-1 (ITLN1) gene highly expressed in visceral fat can inhibit PI3K/Akt pathway in muscle cells through endocrine pathway (60). It has already been mentioned that this pathway is a key signaling axis for myoprotein synthesis, and its inhibition further blocks myosynthesis, forming a cross-organ regulatory pathway from abnormal fat metabolism to myosynthesis inhibition.

The loss of muscle mass is not followed by a quiescent state but by a second hit through prolonged disuse atrophy. The loss of muscle mass leads to decreased mobility, energy expenditure and muscle use frequency, while the “use in and use out” property of muscle will lead to aggravated muscle fiber atrophy (61). This process leads to a further reduction in muscle mass. Consequently, due to the decrease in the number of muscle fibers, the storage of metabolic factors such as carnitine is further reduced, which in turn exacerbates muscle metabolic disorders.

### 3.4 Endocrine dysregulation and metabolic abnormalities

As the core signaling pathway of promoting muscle synthesis and inhibiting muscle decomposition, the functional integrity of Ghrelin-GH-IGF-1 axis directly depends on the endocrine function and inflammatory homeostasis of gastric mucosa. Ghrelin, mainly synthesized by endocrine cells in the gastric mucosa, is a key hormone in regulating appetite. It also stimulates the pituitary gland to release growth hormone (GH) by binding to the growth hormone secretagogue receptor 1a (GHSR1a), thereby activating the IGF-1/mTOR pathway and providing molecular impetus for muscle generation (62). However, in patients with GC, the primary tumor invasion or gastrectomy directly damages the mucosa, leading to a sharp reduction in Ghrelin synthesis (63), cutting off muscle synthesis drive from the signal source. On the other hand, tumor-related inflammation or metabolic reprogramming can reduce the sensitivity of the body to GH, inhibit the expression of GHSR1a, and even inhibit the degradation of insulin-like growth factor binding protein (IGFBP) through proinflammatory factors (64). This seems to be an excessive accumulation of IGFBP to protect IGF-1, but in fact, the amount of free IGF-1 is reduced, which makes it unable to act on muscle cells, and ultimately leads to the overall downregulation of GH-IGF-1 axis function (65).

Disruption of the insulin axis further binds metabolic regulation to muscle mass loss. As the most important anabolic hormone of muscle cells, insulin’s core role is to promote the uptake of glucose and amino acids by muscle cells, providing energy and raw materials for muscle synthesis. But GC traps insulin into functional failure through a dual pathway. Firstly, the abnormal rhythm of gastric emptying after surgery leads to blood glucose fluctuation, insulin secretion is not synchronized with blood glucose changes, and muscle cells are unable to stably obtain energy and are forced to start muscle decomposition (66). In addition, glucose fluctuations together with tumor inflammation induce insulin resistance, making muscle cells less sensitive to insulin. Therefore, it is necessary to simultaneously repair the effect of insulin on muscle cells. Because, even if insulin secretion is normal, it cannot effectively transport nutrients into muscle cells. As the largest glucose storage and utilization organ in the human body, the loss of muscle mass itself will reduce glucose utilization and further aggravate insulin resistance (67).

Hormonal disorders lead to the loss of muscle synthesis, and metabolic imbalance is the collapse of muscle cells. Iron overload in skeletal muscle of patients with GC cachexia leads to direct destruction of muscle cells through oxidative stress damage. The synergistic effect of imbalance of hepcidin-ferroportin axis (inhibition of iron excretion) and up-regulation of divalent metal transporter 1 (DMT1) (promotion of iron intake) leads to abnormal accumulation of iron in muscle cells (68). Excess iron produces a large amount of reactive oxygen species (ROS) through Fenton reaction. However, the levels of antioxidant enzymes such as superoxide dismutase (SOD) and glutathione (GSH) in muscle cells are reduced (69), and uncleared ROS directly damage the membrane structure of muscle cells and activate the apoptotic pathway, leading to active necrosis of muscle cells. The results suggest that there are pathological features of irreversible damage in sarcopenia.

Myosteatosis in the progression of GC is characterized by “loss of subcutaneous fat but increase of intramuscular fat.” Tumors release large amounts of free fatty acids (FFA) by enhancing lipolysis, while muscle cells cannot effectively metabolize FFA due to impaired mitochondrial function, resulting in its deposition in the muscle space and muscle fibers, and lipotoxicity directly destroys the structure of muscle fibers (70). This suggests that fat distribution rather than total fat volume affects muscle mass and provides a new dimension for clinical assessment of the risk of sarcopenia. At the same time, the decreased energy conversion efficiency of abdominal adipose tissue leads to insufficient secretion of anti-inflammatory adipokines, such as adiponectin (60), which could regulate energy metabolism by activating AMPK pathway in muscle cells and regulate muscle differentiation and inflammation through PPAR family (71). Its deletion not only weakens metabolic protection, but also amplifies the effect of increased visceral fat/subcutaneous fat ratio (VAT/SAT) in patients with locally advanced GC (60, 71). Elevated VAT/SAT inhibits fat oxidation, promotes TNF-α/IL-6 releases, and further activates the muscle catabolism pathway.

Amino acids are the key raw materials for the synthesis of skeletal muscle proteins. As mentioned above, GC leads to a deficiency of amino acids in the body through multiple pathways, which in turn causes sarcopenia. However, amino acid supplementation with peripheral intravenous nutrition (PPN) combined with oral high-protein preparations reduced the rate of muscle loss from 6.5 to 4.1% within 7 days after surgery in GC patients (*p* = 0.006) (72). This clinical evidence supports the possible role of insufficient raw materials and also suggests the necessity of precision nutritional supplementation for GC patients. Especially glutamine, as the most abundant non-essential amino acid in muscle, is largely consumed by immune cells and tumor cells after tumor or surgical trauma, making it from “non-essential” to “conditionally essential amino acid” (73). Exogenous glutamine supplementation can reverse this process, promote muscle protein synthesis, and improve psoas muscle atrophy in patients with GC (74).

### 3.5 Mechanisms of treatment-related injury

Although the three core treatment methods for GC (surgery, chemotherapy, and radiotherapy) aim to eradicate or control the tumor, they interact with sarcopenia through multiple pathways. The treatment itself induces or exacerbates the loss of muscle mass, and sarcopenia. In turn, it weakens the patient’s tolerance to the treatment and amplifies its toxicity.

The impact of surgery on sarcopenia begins with traumatic stress and extends to metabolic and microecological disorders. Through tissue injury and ischemia-reperfusion, surgical trauma directly stimulates monocytes to release proinflammatory factors such as IL-6 and TNF-α, which activate the UPS in muscle cells, accelerate muscle fiber degradation, and inhibit IGF-1-mediated mTOR muscle synthesis pathway, leading to metabolic imbalance (75). The stress response triggered by sympathetic nerve stimulation further amplifies this process. Elevated catabolic hormones such as cortisol and glucagon can promote muscle glycogenolysis and enhance gluconeogenesis. Moreover, it directly reduces the efficiency of muscle protein synthesis through hypercortisolemia. And it forces skeletal muscles to release amino acids to meet the needs of energy supply, wound healing and immunity (76). It is of particular concern that the significant disturbance of intestinal flora and the decrease of fecal butyrate level after radical gastrectomy have become “indirect sarcopenia factors” that are easily ignored. As a metabolite of intestinal flora, the reduction of butyric acid will negatively regulate insulin resistance, aggravate systemic inflammation and nutritional metabolism imbalance, and finally delay postoperative muscle function recovery (77), which suggests that intestinal microecology may be a potential regulatory target for surgery-related sarcopenia.

The driving effect of chemotherapy on sarcopenia has the dual characteristics of direct drug toxicity and systemic metabolic interference. About 40–60% of GC patients develop sarcopenia during chemotherapy (78), which is rooted in the direct damage of chemotherapy drugs to muscle cells and the indirect destruction of nutrient absorption. Drugs such as oxaliplatin and fluorouracil destroy muscle mitochondrial function by generating ROS, leading to insufficient energy production and muscle fiber atrophy. At the same time, it interferes with DNA replication of muscle satellite cells and blocks the repair process after muscle injury (79). Chemotherapy-induced systemic inflammation promotes the release of damage-associated molecular patterns (DAMPs) from tumor cells, activate tumor-associated macrophages (TAMs) to release proinflammatory factors, promote muscle breakdown, and inhibit muscle differentiation factors such as MyoD (80). Chemotherapy-related gastrointestinal toxicity (diarrhea, vomiting, mucositis) can reduce energy and protein intake, and more importantly, reduce the expression of L-type amino acid transporter 1 by damaging the intestinal mucosal epithelial cells (81). This transporter is a channel for branched chain amino acids (BCAAs) to enter muscle cells, and its reduced expression leads to the inability of BCAAs to be effectively utilized, further affecting the efficiency of muscle protein synthesis.

Interestingly, corticosteroids (such as dexamethasone) used to alleviate side effects during chemotherapy can reduce nausea and vomiting but activate 11β-hydroxysteroid dehydrogenase 1 (11β-HSD1) in muscle cells, which converts inactive corticosterone into active cortisol and promotes the degradation of muscle protein by binding to glucocorticoid receptors (82). The muscle tissue of patients with sarcopenia has a decreased ability to metabolize chemotherapy drugs, which will lead to a decrease in drug clearance and an increase in blood drug concentration, and then exacerbate chemotherapy toxicity. Toxic reactions destroy the immune barrier and increase the risk of infection, which in turn further activates the inflammatory response to accelerate myolysis (26, 83).

The effect of radiotherapy (RT) on sarcopenia in patients with GC is related to local organ damage. Ionizing radiation released by RT not only damages the gastric mucosa but also affects adjacent organs such as pancreas and intestine. Radiation-related inflammation of the gastrointestinal tract leads to mucosal damage, which directly leads to maldigestion and absorption of myosynthetic substrates (84). Mucosal barrier defects caused by the destruction of intestinal tight junction proteins can induce dysbiosis of flora and endotoxin into the blood, activate systemic inflammatory response and accelerate muscle fiber degradation (85). The pancreas, as a key digestive gland, is particularly affected by radiation dose and volume. Observational study has found a higher incidence of sarcopenia in GC patients receiving higher doses of pancreatic irradiation (86). This is due to radiation damage to pancreatic acinar cells, resulting in reduced trypsin and lipase secretion, which further impaired the digestion and resorption of muscle synthetic raw materials. Prospective dose–response studies are needed for further confirmation. In addition, the direct effects of RT on muscle tissue are often underestimated. Radiation damage to vascular endothelium leads to muscle tissue ischemia and hypoxia, activates the hypoxia-inducible factor-1α (HIF-1α) pathway to up-regulate catabolic enzymes, and blocks the nutrient supply of muscle stem cells, leading to muscle fiber repair disorders and persistent atrophy (87).

In summary, sarcopenia in GC arises from the synergistic effects of multiple pathological mechanisms that interact and reinforce one another, ultimately forming a vicious cycle between GC and sarcopenia. Table 3 systematically integrates clinically validated interventions and exploratory therapeutic strategies corresponding to distinct pathological mechanisms. These mechanistic insights establish a critical framework for designing multimodal management regimens, which serve as core approaches to ameliorating sarcopenia, optimizing GC patient prognosis, and guiding the subsequent exploration of individualized intervention strategies.

**Table 3:** Overview of pathological mechanisms and corresponding management strategies for sarcopenia in GC.

Mechanism category	Key pathological mechanisms	Targeted management strategies	Potential solutions	References
Inflammation-mediated myostasis imbalance	Pro-inflammatory cytokines (IL-6, TNF-α, IL-8) activate JAK/STAT3 and NF-κB pathways UPS/ALP are activated to accelerate muscle protein degradation The expression of protective muscle factor FSTL1 is decreased	Preoperative immune enteral nutrition with *ω*-3 fatty acids and arginine Moderate-intensity exercise to induce anti-inflammatory cytokines Exploratory use of anti-inflammatory drugs	Targeted use of celecoxib to regulate oxidative muscle fiber formation via PROKR1 pathway Inhibition of JAK/STAT3/NF-κB signaling to block muscle protein degradation pathways	(35, 36, 40, 105, 116, 128)
Immune function impairment	Muscle mass loss leads to insufficient nutrient substrates for immune cells Sarcopenia combined with high IL-16 expressions recruit immunosuppressive cells Downregulated muscle-derived SPARC weakens immune surveillance Tumor-derived C/EBPβ induces secretion of muscle atrophy factors and inhibits immune cell activation	Perioperative glutamine supplementation Monitor CCR during PD-1 inhibitor therapy Resistance exercise to enhance immune cell infiltration	Enhancement of muscle-derived SPARC expression to strengthen immune surveillance and suppress myeloid-derived suppressor cells Exercise-induced promotion of tertiary lymphoid structure formation in tumor tissues	(42, 44–46, 48, 74)
Malnutrition & energy metabolism disorder	Tumor obstruction, cancer pain, and hypothalamic appetite suppression reduce nutrient intake Gastrectomy causes malabsorption of protein, iron, and vitamins Tumor warburg effect sequesters glucose/amino acids and inhibit muscle mitochondrial function Carnitine deficiency impairs muscle fatty acid oxidation visceral fat ITLN1 inhibits the PI3K/Akt pathway Muscle loss induces disuse atrophy and exacerbates metabolic disorders	Preoperative 10–14 days high-protein ONS with HMB Early postoperative enteral nutrition with short-peptide formula Supplement vitamins, trace elements and carnitine to improve muscle energy metabolism	Targeted regulation of visceral fat intelectin-1 to restore PI3K/Akt pathway activity for muscle protein synthesis Intervention of tumor Warburg effect to reduce glucose/amino acid sequestration and protect muscle mitochondrial function	(52, 54, 56, 58, 103, 107, 112)
Endocrine & metabolic dysregulation	Gastric mucosa damage reduces Ghrelin synthesis, downregulating the Ghrelin-GH-IGF-1 axis Postoperative blood glucose fluctuation and insulin resistance impair muscle nutrient uptake Muscle iron overload generates ROS via the Fenton reaction to damage muscle cells Intramuscular fat accumulation causes lipotoxicity Reduced adiponectin secretion amplifies inflammation and muscle catabolism Systemic amino acid deficiency limits muscle protein synthesis	Administration of ghrelin receptor agonist anamorelin to activate the Ghrelin-GH-IGF-1 axis and promote muscle anabolism Individualized metformin administration Iron chelator therapy targeting hepcidin-ferroportin axis modulation Perioperative amino acid supplementation	Restoration of gastric mucosa endocrine function to increase ghrelin synthesis Regulation of hepcidin-ferroportin axis to reduce muscle iron accumulation Promotion of adiponectin secretion to inhibit intramuscular fat deposition and lipotoxicity	(62, 64, 67, 68, 126)
Antitumor therapy-related injury	Surgical trauma induces stress response and intestinal flora dysbiosis Chemotherapy drugs generate ROS to damage muscle mitochondria and satellite cells Gastrointestinal toxicity reduces BCAA transport Glucocorticoids promote muscle protein degradation Radiotherapy damages gastrointestinal/pancreatic function and muscle vascular endothelium	Preoperative home-based prehabilitation Dynamic SMI monitoring with personalized chemotherapy dosage adjustment Early postoperative mobilization Neuromuscular electrical stimulation to prevent disuse atrophy Post-discharge ONS combined with dietary guidance	Modulation of intestinal flora to increase butyrate production and accelerate postoperative muscle function recovery Use of antioxidants to mitigate chemotherapy-induced muscle mitochondrial damage Optimization of radiotherapy dose-volume parameters to reduce pancreatic and gastrointestinal mucosa injury	(75, 77, 79, 117, 120, 123)

GC, gastric cancer; IL, interleukin; TNF-α, tumor necrosis factor-α; JAK/STAT3, janus kinase/signal transducer and activator of transcription 3; NF-κB, nuclear factor-kappa B; UPS, ubiquitin-proteasome system; ALP, autophagy-lysosome pathway; FSTL1, follicle-statin-like protein 1; ω-3, omega-3 polyunsaturated fatty acids; PROKR1, prokineticin receptor 1; CCR, creatinine/cystatin C ratio; PD-1, programmed cell death protein 1; SPARC, secreted protein acidic and rich in cysteine; C/EBPβ, CCAAT/enhancer-binding protein β; ONS, oral nutritional supplements; HMB, β-hydroxy-β-methylbutyrate; PI3K/Akt, phosphoinositide 3-kinase/protein kinase B; Ghrelin-GH-IGF-1 axis, ghrelin-growth hormone-insulin-like growth factor 1 axis; ROS, reactive oxygen species; BCAA, branched-chain amino acid; SMI, skeletal muscle index; NMES, neuromuscular electrical stimulation.

## 4 Multimodal management of sarcopenia in GC

### 4.1 Pre-treatment evaluations and identifications of high-risk individuals

In the diagnosis and treatment of gastric cancer, sarcopenia is a key variable affecting prognosis. Precise assessment before treatment and identification of high-risk groups, by analyzing muscle status and function to overall nutritional status, providing a basis for individualized intervention and adjustment of treatment plans. To achieve the synergistic management of sarcopenia during the process of anti-tumor treatment. The ESPEN guidelines strongly recommend the use of the GLIM (Global Leadership Initiative on Malnutrition) criteria as the diagnostic framework for malnutrition (including sarcopenia) (88). GLIM combines phenotypic and etiological indicators, forming a three-dimensional consensus on muscle mass, muscle strength and muscle function, which is more suitable for cancer patients.

Skeletal muscle index (SMI) was used as the gold standard for the assessment of muscle status, which was calculated by measuring skeletal muscle cross-sectional area at the third lumbar spine (L3) level by preoperative abdominal computed tomography (CT), combined with height standardization, and using sex-specific thresholds (e.g., <52.4cm^2^/m^2^ or 45.4cm^2^/m^2^ for males; <38.5cm^2^/m^2^ or 34.4cm^2^/m^2^ for women) (89). The diagnostic thresholds are different in different regions and populations, and the validation criteria in our center or region should be given priority. This also shows the urgency of unifying diagnostic criteria. As a supplement, skeletal muscle density (SMD) can reflect the degree of intramuscular fat infiltration by CT. Low SMD is an independent risk factor for poor prognosis of GC after surgery (90). This suggests that even if SMI is normal, increased intramuscular fat may still affect the prognosis. In addition, dual-energy X-ray absorptiometry (DXA) is suitable for the assessment of whole-body muscle composition (9), bioelectrical impedance analysis (BIA) is simple but easily affected by body fluid status, and it needs to be combined with CT to verify (91).

Muscle function assessment to focus on individual muscle mass and functional matching. Classical indicators such as hand grip strength (HGS) (<28 kg for men and <18 kg for women) and gait speed test (GST) (<0.8 m/s) can initially determine functional status (92). But stair climbing test (SCT) has more clinical value and provides a more suitable choice for preoperative rapid screening. It not only reflects the muscle strength of the lower limbs but also is significantly correlated with muscle state indicators. Low SCT performance (SCT time ≥12.65 s) is better than GST in predicting the prognosis and complications after GC surgery (93).

Nutritional inflammation indicators explain the causes of sarcopenia and supplement risk information from the systemic level. Prognostic nutritional index (PNI) is based on serum albumin and lymphocyte count, which directly reflects nutritional reserve and immune status of patients with GC (94). Nutritional risk screening 2002 (NRS 2002) is widely used in patients with GC through disease and nutritional impairment score (≥3 points indicate risk) (95). Inflammatory indicators such as modified Glasgow Prognostic Score (mGPS) and neutrophil/lymphocyte ratio (NLR) are closely related to the risk of muscle loss and survival time because chronic inflammation can accelerate muscle degradation and inhibit muscle synthesis (96). Serum ferritin, phase angle (PhA), cholinesterase (CHE) and urinary titin N-terminal fragment (UTF) can also be used as supplementary evaluation indicators to provide more basis for comprehensive judgment of the patient’s physical status before surgery and avoid missing sarcopenia patients with a single indicator (91, 97, 98).

**Figure 2:**
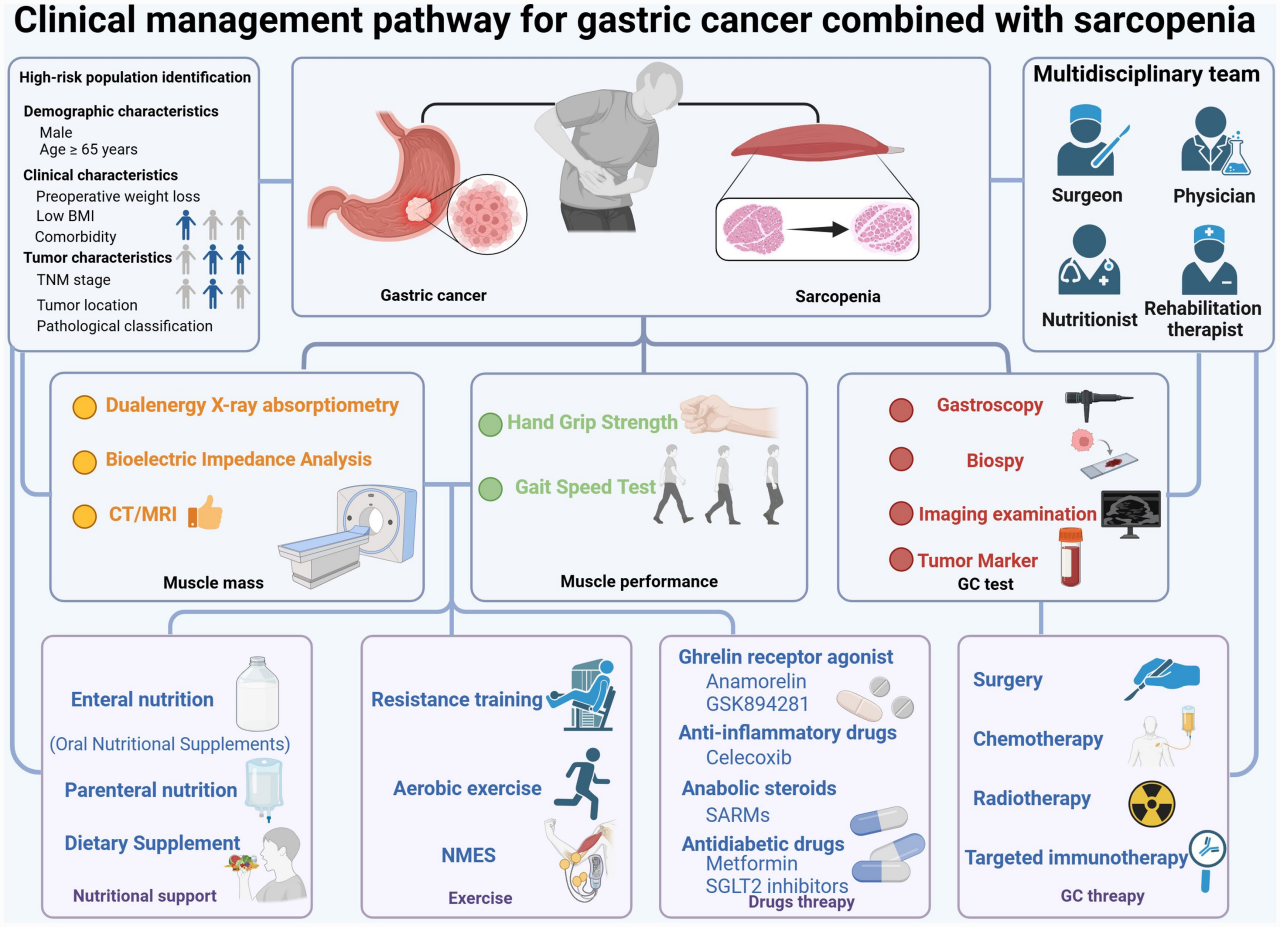
Multidisciplinary management pathway for sarcopenia in patients with GC. This illustration describes the clinical management pathway for GC complicated with sarcopenia, including comprehensive assessment of muscle mass and muscle performance, as well as GC-specific diagnosis. Subsequent comprehensive intervention measures include nutritional support, exercise programs, drug treatment, and standard GC therapy. Throughout the process, a multidisciplinary team is required to participate, and high-risk population identification and individualized diagnosis and treatment plans should be formulated. It should be noted that this figure is only a recommended framework for clinical management and is not a standardized clinical guideline. BMI, body mass index; CT, computer tomography; GC, gastric cancer; NMES, neuromuscular electrical stimulation; MRI, magnetic resonance imaging; SARMs, selective androgen receptor modulators; SGLT2, sodium-dependent glucose transporters 2. Created in BioRender. Liu, W. (2026) https://BioRender.com/9lxwvli.

The identification of high-risk groups require comprehensive judgment based on demographic, clinical and tumor characteristics. Elderly patients over 65 years old (especially over 75 years old) and male patients have a high incidence of sarcopenia and a poor prognosis (17, 99). Patients with preoperative weight loss, low BMI and underlying diseases such as type 2 diabetes mellitus have an increased risk of sarcopenia and postoperative complications (100, 101). The incidence of sarcopenia in GC patients with TNM stage III–IV is higher than that in the early stage, and the prognosis is worse (102). In addition, as mentioned above, different subprimary sites or different surgical methods have significant differences in the incidence and prognosis of sarcopenia. This should also be given due attention when identifying high-risk patients (14, 56). A multidisciplinary management pathway for sarcopenia in GC patients, encompassing assessment, diagnosis, and intervention, is proposed in Figure 2.

### 4.2 Interventions and treatment measures

#### 4.2.1 Nutritional support therapies

Patients with GC often have malabsorption of nutrition due to tumor consumption, and anti-tumor comprehensive treatment. Nutritional support is the primary measure to prevent and improve sarcopenia by supplementing protein, energy and key nutrients to correct the deficiency of muscle synthesis substrates. The plan should be adjusted according to the treatment stage for GC.

Preoperative nutritional pretreatment was used as the starting point for reversing muscle loss. The guidelines of the European Society for Clinical Nutrition and Metabolism (ESPEN) clearly pointed out that nutritional intervention for patients with sarcopenia can effectively reduce postoperative muscle breakdown and improve clinical outcomes (88). This definition emphasizes that nutritional status is an important prerequisite for surgical safety. Specific intervention strategies should be stratified according to patients’ oral capacity. For patients capable of oral intake, the ESPEN guidelines recommend at least 10 to 14 days of nutritional supplementation, even if this means that the surgery needs to be postponed (88). In addition, Yamamoto et al. (103) suggested that high-protein high-energy oral nutritional supplements (ONS) containing β-hydroxyl-β-methylbutyric acid (HMB) be administered 1 to 4 weeks preoperatively. Among them, HMB, as a key regulator of muscle protein synthesis, can achieve the reversal of 18.2% of patients from the state of sarcopenia to the state of non-sarcopenia (103). For patients who are unable to eat orally or have insufficient tolerance, enteral nutrition (EN) support via a nasojejunal tube or short-term parenteral nutrition (PN) is recommended, especially for those with hypoalbuminemia (<35 g/L) (104). These nutritional support methods help maintain intestinal barrier function, improve sarcopenia in elderly GC patients, and at the same time lower the risk of postoperative abdominal infection. Preoperative nutritional intervention can also enhance the body’s tolerance to surgical trauma, play a buffer role and reduce the risk of surgery by regulating the immune and inflammatory state of the body. Compared with standard EN, the total postoperative complication rate (28.6%) of immune enteral nutrition (EIN) containing *ω*-3 fatty acids, arginine and nucleotides 7 days before surgery was significantly lower than that of counterpart (44.6%, *p* = 0.049) (105).

Postoperative nutritional support focused on muscle restoration and treatment tolerance improvement. Initiation of EN within 24 to 48 h after surgery is critical. Although intestinal function is weak at this time, early EN can be infused with hypotonic formula such as short peptides through nasojejunal tube or jejunostomy. While reducing the risk of intestinal barrier failure, it also provides an early substrate for muscle recovery (106). A prospective study involving 76 patients undergoing radical gastrectomy confirmed that perioperative EN intervention (preoperative nutritional preparation + stepwise EN infusion within 24 h after surgery) reduced the rate of skeletal muscle loss to 3.5% at 7 days after surgery, which was approximately 50% lower than the control group (7.1%) (*p* < 0.01) (107). At the same time, grip strength loss was reduced by 25% and weight loss was improved by 27% (107), which directly reflected the effectiveness of the whole nutrition management. It should be noted that a retrospective analysis of 83 patients undergoing radical surgery showed that muscle loss was significantly reduced in the amino acid supplementation group 7 days after surgery, but there was no statistically significant difference in muscle loss at 1 month after surgery (72). This finding reveals the short-term effectiveness and long-term deficiency of postoperative nutritional intervention and suggests the importance of nutritional continuation after discharge. For patients with nutritional risk who have an NRS 2002 score of ≥3, providing ONS combined with dietary guidance after discharge can increase the SMI and reduce the incidence of sarcopenia (108). It can also improve chemotherapy tolerance by alleviating symptoms such as fatigue and loss of appetite. This provides a partial guarantee for comprehensive anti-tumor treatment.

Compared with deliberate nutritional supplementation, long-term nutrient supplementation in daily diet is the basic guarantee to prevent the recurrence of sarcopenia. Its core lies in “evidence-based selection” rather than blind supplement. Studies have confirmed that a variety of natural products can protect skeletal muscle health through multi-target synergistic mechanisms, such as regulating the expression of myokines, activating antioxidant pathways, and inhibiting protein degradation systems (109). However, when it is specific to a single nutrient, it is necessary to prioritize based on the strength of evidence. Vitamin D is hydroxylated twice through skin and kidney to produce the active metabolite calcitriol, which can regulate calcium reflux of muscle cells and enhance mitochondrial function through Akt/mTOR pathway (110). The 1-year supplement study showed that it could significantly improve muscle mass (1.9% vs. −3.4%) and muscle strength (4.1% vs. −0.7%) (111), which provided a clear intervention direction for elderly GC patients with a high incidence of vitamin D deficiency. In addition, a systematic review covering 45 studies pointed out that selenium and magnesium have moderate quality evidence to support the improvement of muscle mass, muscle strength and the reduction of the sarcopenia’s incidence in middle-aged and elderly people (112), while the evidence for potassium, iron, sodium, phosphorus and other minerals is not clear due to insufficient research (112). This difference indicates that daily nutritional supplementation needs to be precisely regulated rather than blindly excessive. The latter will increase the metabolic burden on the body, making the patient lose more than they gain.

#### 4.2.2 Exercises and physical rehabilitations

Preoperative exercise intervention can improve patients’ tolerance to surgical trauma through early physical energy reserve and muscle mass maintenance. As the core form of such therapy, resistance exercise can directly activate the muscle protein synthesis pathway by stimulating the contraction of muscle fibers in the range of medium and low intensity, and its effect is positively correlated with exercise intensity (113). Aerobic exercise can improve metabolic endurance by increasing maximum oxygen uptake and enhancing mitochondrial oxidase activity (114), and the combination of the two can synergistically optimize the efficiency of muscle synthesis (115). More importantly, regular exercise can also induce the body to secrete anti-inflammatory cytokines, which mechanistically inhibit muscle atrophy mediated by cancer-related inflammation (116). Clinical evidence further validates the value of preoperative exercise. A randomized controlled study involving 128 patients with GC showed that the 6-minute walk distance of the home prehabilitation group (assigned 1:1 with the standard care group) increased by 31 meters before surgery, the incidence of postoperative complications and the non-compliance rate of neoadjuvant therapy were reduced (117). Another trial involving 26 patients with locally advanced esophagogastric junction cancer (receiving 15 weeks of continuous systemic exercise before surgery) found that the intervention group had a smaller decline in peak oxygen uptake (peak VO_2_) and less muscle loss compared to 28 patients in the control group (118).

Postoperative exercise intervention not only focuses on short-term muscle mass restoration after surgery but also attaches importance to long-term muscle function maintenance to prevent sarcopenia recurrence. Passive or mild active bedside exercise should be initiated 1–3 days postoperatively, with a gradual transition to ambulation and resistance training (119). This “early mobilization” strategy can accelerate the recovery of gastrointestinal function and also inhibit the disuse atrophy of muscle fibers through early mechanical stimulation, thereby saving muscle repair time. A prospective study of 18 GC patients over 65 years old with gastrectomy confirmed that moderate intensity walking and resistance training with branched chain amino acids (BCAAs) supplement after surgery could control the decrease of SMI to 4.6% at 1 week after surgery and further narrow to 2.1% at 1 month (120). And the quality of life (QOL) was close to the preoperative level. It directly reflects the strong effect of exercise combined with nutrition collaborative intervention. Regular exercise during long-term follow-up is also critical. A survey of healthy nutritional status of 338 patients with GC showed that the participation rate of strength training in patients diagnosed ≥5 years was significantly lower than that in healthy control group (OR = 0.548), especially in male patients over 65 years old (121). This data revealed the problem of insufficient long-term exercise compliance among postoperative patients with GC and also suggested that clinical practice should strengthen long-term exercise education to avoid a gradual decrease in muscle during follow-up. Currently, a clinical study involving 324 patients is being carried out in South Korea to evaluate the effects of exercise and individualized rehabilitation programs monitored by wearable devices on physical fitness and quality of life of postoperative patients within 1 year (122). This study is expected to provide technical support for the precise management of postoperative exercise intervention for GC.

For patients who are unable to carry out active exercise due to severe illness, extreme physical weakness or movement disorders, passive stimulation therapy becomes an important supplement to exercise intervention, which effectively covers the clinical scenarios that are unable to carry out active exercise. Neuromuscular electrical stimulation (NMES), which can be used alone or in combination with active activities, can stimulate muscle motor points through surface electrodes to trigger contraction (123). The whole-body electrical stimulation (WB-EMS) technology developed in recent years can stimulate the main muscle groups of trunks and limbs at the same time, which has been proved to improve the muscle strength of elderly patients with sarcopenia (124). The unique advantage of EMS lies in the non-selective recruitment of type I (slow muscle) and type II (fast muscle) muscle fibers. Even if patients are unable to actively exert force, they can still maintain muscle fiber activity through electrical stimulation, and studies have shown that even if some effects fade after stopping stimulation, patients’ muscle strength and muscle cross-sectional area can still be maintained above the level before training (123).

#### 4.2.3 Pharmacological interventions and emerging therapies

Drug interventions focus on improving muscle synthesis and reducing inflammation, while considering anti-tumor properties. At present, most of them are in the exploratory stage. Some drugs have shown great potential in specific populations.

Anamorelin, which targets the Ghrelin-GH-IGF-1 axis, is one of the few drugs that have been clinically transformed, providing a clear paradigm for the intervention of the myosynthetic pathway. As an oral Ghrelin receptor agonist, it increased gastrocnemius muscle weight by 18% and food intake by 25% in a mouse model by activating this pathway (125). This result not only validates the central role of the Ghrelin axis in muscle regulation but also lays the foundation for clinical studies. Although it has not been approved in Europe and the United States, Japan used it for the treatment of cancer cachexia (including GC) in 2020, becoming the first oral drug for this indication in the world (126), filling the gap of targeted therapy for GC combined with sarcopenia. A recent prospective study in Japan involving 229 patients with GC cachexia further confirmed that after 12 weeks of anamorelin treatment, 83 patients gained significant weight, some patients’ appetite improved, and the overall tolerance was good (64). However, it should be noted that it mainly improves outcomes such as weight and appetite, but the muscle function regulation and survival improvement of GC patients are still unclear. There are still differences in efficacy and tolerance among different populations. Future large randomized controlled trials are needed to define the optimal benefit in the population and to define the scope of application.

Non-steroidal anti-inflammatory drugs (NSAIDs), as classical anti-inflammatory drugs, theoretically play an auxiliary role in anti-tumor therapy by inhibiting chronic inflammation and relieving myolysis, but they are in trouble in clinical practice. A systematic review of cachexia in adult cancer (including GC) showed that there was insufficient evidence to prove the efficacy and safety of indomethacin and ibuprofen, and it was difficult to determine the application value of both in GC complicated with sarcopenia (127). Although celecoxib is well tolerated in patients with GC at doses of 200–400 mg/day, the efficacy results are inconsistent, and the use of celecoxib alone cannot be clearly recommended (127). However, basic research provides a new direction for the “old drugs for new use.” Experiments in mice have found that celecoxib can significantly promote the formation of oxidative muscle fibers, enhance mitochondrial function and fatty acid oxidation capacity by targeted activation of PROKR1 pathway (128), and effectively improve the decline of muscle function and metabolic disorders induced by high-fat diet. This mechanism suggests that it may directly exert muscle protection by regulating muscle cell metabolism. Overall, the evidence for NSAIDs in the treatment of gastric cancer complicated with sarcopenia is insufficient and highly heterogeneous, and their routine use is not recommended. In the future, specific clinical trials for GC patients need to be designed to accurately evaluate their value.

Although androgen drugs (such as testosterone supplements) can increase muscle mass and strength, especially for the elderly with low basal testosterone levels, cardiovascular and prostate risks limit their application (129), and modern strategies emphasize individualized drug delivery. Selective androgen receptor modulators (SARMs) have shown the potential to improve physical performance and increase lean mass in phase II clinical trials with the advantages of high tissue targeting and few systemic side effects (130). However, SARMs have not been approved by the FDA for GC patients, and their safety in the tumor microenvironment still needs to be verified.

Anti-myostatin peptide has improved muscle mass and inflammatory state in animal models, but the results of human clinical trials are mixed, and some patients have terminated the study due to severe side effects (131). Its related factor GDF11 also leads to controversial conclusions due to defective protein preparation (130), reflecting the difficulty in translational medicine. At present, there is a lack of research on GH in patients with GC combined with sarcopenia. Moreover, systematic review has pointed out that although GH increases muscle mass in people without specific diseases, it is accompanied by a large number of treatment-related adverse reactions, leading to a high withdrawal rate from clinical trials (132). In contrast, the new generation of growth hormone secretagogues and Ghrelin receptor agonists have become more promising directions due to their better efficacy and fewer side effects.

As an “accidental discovery” of muscle protection potential drugs, anti-diabetic drugs have become a class of drugs with great prospects. Among insulin sensitizers, metformin significantly inhibits the expression of dystrophin by activating AMPK pathway, and clinical studies have shown that it can improve the lean body mass/fat ratio in patients with type 2 diabetes mellitus (T2DM), but high doses may weaken exercise-induced muscle mitochondrial fitness (133). For GC patients who often require exercise intervention, it is necessary to determine the optimal therapeutic dose. Thiazolidinediones (TZDs) present a paradox. Pioglitazone enhances muscle energy metabolism, whereas rosiglitazone is associated with reduced thigh muscle mass (133). It is suggested that the structural differences of the same kind of drugs may lead to opposite muscle metabolic effects. The myoprotective effect of insulin secretagogues is mainly negative (133), which limits their application.

Although the new generation of GLP-1 receptor agonists have shown potential in the management of obesity (134), clinical studies have found that semaglutide may lead to the decline of muscle strength and physical function by causing neuromuscular junction degeneration and neuronal damage (135). However, the net benefit in patients with GC complicated with sarcopenia remains unclear and requires individualized assessment and awaits prospective studies. Moreover, cohort studies have shown that the risk of GC in patients with T2DM is higher for GLP-1 receptor agonists than for SGLT-2 inhibitors (135). This is a key safety concern for patients with GC. Therefore, although SGLT-2 inhibitors, which are also a new generation of anti-diabetes drugs, may play a positive role in patients with T2DM complicated with GC, whether they can maintain muscle mass and strength and their safety in practical application still need to be verified by special studies on GC population.

## 5 Conclusion and future directions

Sarcopenia acts as a pivotal adverse factor in gastric cancer (GC) progression and management, exerting far-reaching impacts throughout the disease course. GC accelerates muscle wasting via chronic inflammation, metabolic/nutritional disorders, and therapeutic injury, while sarcopenia impairs anti-tumor immunity, diminishes treatment tolerance, and exacerbates GC progression. This interplay directly leads to poor prognosis such as increased postoperative complications, enhanced chemotherapy toxicity and shortened survival, highlighting the necessity of integrating sarcopenia management into GC clinical care. Current multimodal interventions, including stratified nutritional support, stage-specific exercise rehabilitation and exploratory pharmacotherapies such as ghrelin receptor agonists, have shown preliminary effects in alleviating muscle loss and improving outcomes. However, critical gaps persist, including inconsistent regional diagnostic criteria for sarcopenia, limited evidence supporting most pharmacotherapies, and a lack of long-term personalized management strategies.

The focus in the future is to standardize the diagnostic criteria for sarcopenia and address the regional applicability differences of the existing diagnostic criteria. Through multi-center studies, muscle mass, function and inflammatory indicators were integrated to develop rapid screening tools and establish three-dimensional stratified diagnosis. Deepening mechanism research on muscle fiber damage caused by abnormal body metabolism, absorption and utilization, and the bidirectional regulatory network of myokines. Focusing intervention strategy innovation on precision nutrition, construction of intelligent exercise rehabilitation systems and exploration of combined application of drugs, nutrition and exercise. Shifting clinical management to individualized and long-term models to finally realize the transformation from disease treatment to whole-course health management.
